# Analysis of status quo and influencing factors of personal mastery of patients after fracture surgery

**DOI:** 10.3389/fpsyg.2025.1635147

**Published:** 2025-11-11

**Authors:** Qianqian Zhou, Hongying Wu, Shenying Ji, Xiu Li, Yiwen Jiang, Jie Xia

**Affiliations:** 1 School of Nursing and Health Management, Shanghai University of Medicine and Health Sciences, Shanghai, China; 2 Wusong Central Hospital, Shanghai, China; 3 Nursing Department, Shanghai Pudong New Area Heqing Community Health Service Center, Shanghai, China

**Keywords:** personal mastery, fracture patient, influencing factors, social support, self-efficacy

## Abstract

**Background:**

Effective management of psychological stress is very important for the rehabilitation process after fracture surgery. The purpose of this study is to explore the current status and factors influencing the sense of personal mastery in patients following fracture surgery and provided insights for enhancing patients’ personal mastery to foster positive coping mechanisms in response to sudden health challenges.

**Methods:**

From November 2023 to February 2024, patients who underwent fracture surgery at a tertiary level-A hospital in Shanghai were recruited. The general information questionnaire, personal control scale, general self-efficacy scale, and perceived social support scale were used for questionnaire survey.

**Results:**

One hundred thirty-one patients after fracture surgery in this study. The mean score of personal control was (25.02 ± 6.3). The results of multiple linear regression analysis showed that family residence, marital status, postoperative time, self-efficacy and perceived social support were the influencing factors of patients ‘sense of personal control after fracture surgery (*p* < 0.05).

**Conclusion:**

The sense of personal control of patients after fracture surgery was at a medium level. Married patients, families living in cities and towns, the longer the postoperative time, the higher the level of self-efficacy of fracture patients, the stronger the sense of personal control. Nurses should develop targeted care plans to effectively enhance the patient’s dependence, but also to improve their sense of personal control, to get a better life experience.

## Introduction

A fracture is defined as the disruption of bone integrity and continuity. While fractures are primarily caused by mechanical injuries, other factors such as bone diseases, car accidents, explosions, falls, and osteoporosis may also contribute to their occurrence. Fractures are often accompanied by damage to the surrounding soft tissues. Due to the prolonged healing process, some patients may develop complications such as wound infections, limb dysfunction, joint stiffness, and pressure sores (Van Bergen et al., 2023). These complications typically require extended periods of treatment and recovery, significantly affecting patients and their families (Krakers et al., 2024). Blasco et al. (2012) demonstrated that most patients with traumatic fractures can achieve the effect of fracture healing within 6 months post-surgery, regardless of receiving conservative treatment or internal and external fixation. However, partial functional loss, decreased self-care abilities, and uncertainty about the future can cause patients to experience certain challenges postoperatively, such as greater psychological pressure and obvious negative emotions—including anxiety, depression, and fear—which hinder their postoperative functional exercise and rehabilitation efforts (Bian et al., 2024). Therefore, effective management of psychological stress is critical to the rehabilitation process following fracture surgery. It is of great value to help patients change their coping styles—both cognitive and behavioral—and explore the various positive coping mechanisms in rehabilitation, which can alleviate negative emotions and significantly improve treatment outcomes.

Personal mastery refers to an individual’s ability to manage or control their life, enabling them to better cope with stress, recognize potential threats, and effectively mobilize internal and external resources to aid in postoperative rehabilitation (O’Connor et al., 2021). One study (Raaijmakers et al., 2014) proposed that sufficient personal mastery plays a pivotal role in measuring a patient’s disease self-management capabilities and quality of life in advance. Serving as a mediating variable between social support and quality of life, personal mastery is critical for maintaining patients’ physical and mental health. While previous research examined personal mastery in patients undergoing renal transplantation, coronary heart disease treatment, peritoneal dialysis, and breast cancer care (Diao et al., 2024), only limited evidence concerning the sense of personal mastery in patients after fracture surgery is available. Therefore, this study aims to enhance patients’ sense of personal mastery and improve their overall quality of life by understanding the current state of their personal mastery following fracture surgery and analyzing its influencing factors.

## Materials and methods

This study was conducted in accordance with the Strengthening the Reporting of Observational Studies in Epidemiology (STROBE) guidelines.

### Design and sample

This cross-sectional study was conducted from November 2023 to February 2024. Patients with fractures who underwent surgical treatment in the Department of Orthopedics and Traumatology at a tertiary level-A hospital in Shanghai were selected using convenience sampling. A tertiary level-A grade hospital complies with China’s current “Hospital Classification and Management Measures,” etc. Tertiary level-A hospitals represent the highest category of hospitals, apart from national specialist hospital.

### Inclusion criteria

(a) Age ≥ 18 years; (b) A clinical diagnosis of a fracture; (c) Successful surgical treatment with recovery from anesthesia; and (d) Clear consciousness with the ability to communicate with people and fill in the questionnaire independently. Five patients voluntarily participated in the pilot survey with informed consent.

### Exclusion criteria

Patients with severe lung, heart, kidney, liver, or other diseases, and (b) Patients diagnosed with mental illness. The sample size was determined based on Malone et al. (2016), which recommends 5–10 times the number of independent variables, with an additional 20% accounted for efficiency. As a result, the final required sample size was estimated at 118 participants.

Ultimately, a total of 131 patients who underwent fracture surgery was included in this study. The study was approved by the hospital ethics committee, and all participants provided informed consent prior to enrollment.

### Survey tools

- A general information questionnaire was designed by the researchers themselves to collect general demographic data, such as sex, age, education level, family residence, caregiver availability, marital status, and medical insurance type. Disease-related data, such as fracture cause, fracture site, and postoperative time, was also recorded.
- The personal mastery scale (PMS), originally developed by Pearlin based on a large number of cases and data, was used to assess a patient’s subjective perception of their surrounding environment, personal events, and sense of mastery. The scale comprises seven items rated using the Likert 5-level scoring method, with total scores ranging from 7 to 35 points. Higher scores indicate as stronger sense of control. The Chinese version of the Yu scale (Diao et al., 2024) was employed in this study, with a Cronbach’s alpha coefficient of 0.810 and a retest reliability of 0.63.
- The perceived social support scale (PSSS) represents a positive emotional experience where it measures an individual’s perceived support from their social environment and relationship network through non-quantifiable subjective characteristics (Iovino et al., 2025). Compiled by Zimet et al. (1990), the scale includes 12 items divided across three dimensions: family, friends, and other support. The Likert 7-level scoring method was used to rate each item according to the following scale: “very strongly disagree, very disagree, slightly disagree, neutral, slightly agree, very agree, very strongly agree.” The first level is one point, and each level increases by one point. Total scores range from 12 to 84 points. Higher scores reflect higher levels of perceived social support. In this study, Cronbach’s alpha coefficient for the PSSS was 0.880.
- The general self-efficacy scale (GSES), which assesses an individual’s degree of confidence in overcoming challenges or dilemmas, directly affecting their behavior, was also determined (Drenkard et al., 2022). The scale consists of 10 items scored using a Likert 4-level scoring method. The first level is one point, and each level increases by one point, with total scores ranging from 10 to 40 points. The higher the score, the higher the patient’s self-efficacy level (Sun et al., 2021). In this study, Cronbach’s alpha coefficient for the GSES was 0.870.

### Data collection and analysis

A questionnaire was provided to patients who met the inclusion criteria within the Department of Orthopedics and Traumatology. The investigators used standardized instructions to guide participants in completing the questionnaire. A total of 135 questionnaires were distributed and returned. Upon screening, four were excluded on the grounds that the respondents uniformly selected the same response option for all items, leaving 131 valid questionnaires for the final analysis, yielding an effective response rate of 97%. All statistical analyses were performed using SPSS version 27.0. Measurement data conforming to a normal distribution were expressed as means and standard deviations, and comparisons were performed using independent sample *t*-tests and analysis of variance. Categorical data were expressed as frequencies and percentages. Factors influencing personal mastery in patients following fracture were analyzed using various tests, such as the independent sample *t*-test, univariate analysis, Pearson correlation coefficient, and multiple linear regression analysis. Statistical significance was set at *p* < 0.05.

### Ethical considerations

This study was conducted in accordance with the principles of the Declaration of Helsinki and was approved by the Ethics Review Committee of the University (Approval Number: 2024-bkskt-02-51030420000911672X).

## Results

### Personal mastery scores of patients after fracture surgery

The total PMS scores for patients after fracture surgery was 25.02 (6.3). Among all items, “I can do almost anything I want to do” had the lowest score at 2.76 (1.11) points. The scores for each PMS item are presented in Table 1.

**Table 1:** Scores of each item of personal mastery in patients after fracture surgery (*n* = 131).

Variables	Mean (SD)
1. What my future will be depends mainly on myself.	3.01 (1.45)
2. I feel helpless when dealing with the problems in life.	2.83 (1.08)
3. I can hardly change many important problems in life.	2.80 (1.04)
4. I can’t solve the problems in my life.	2.93 (1.08)
5. I can hardly control what happens to me.	2.80 (1.15)
6. I feel that my life is beyond my control.	2.86 (1.00)
7. I can do almost anything I want to do.	2.76 (1.11)
Aggregate score	25.02 (6.30)

### Comparison of personal mastery scores among patients with different characteristics after fracture surgery

Table 2 provides an overview of the demographic characteristics of the participants. The results revealed that the male-to-female ratio was close to 1:1, with most of them being married (*n* = 112, 85.5%) and residing in urban areas (*n* = 72, 55%). Seventy-five of them had high school education or higher (57.2%), and 88 patients had health insurance (67.2%). The cause of fracture in the vast majority of patients was direct violence (*n* = 116, 88.5%), and the caregiver was predominantly the spouse (*n* = 100, 76.3%). The majority of patients (*n* = 54, 41.2%) were in the early postoperative period (1–3 days). One-way ANOVA revealed statistically significant differences in PMS scores with respect to sex, education level, family residence, marital status, and postoperative duration.

**Table 2:** Comparison of personal mastery among patients with different social demographic characteristics after fracture surgery (*n* = 131).

Variables	*n* (%)	Mean (SD)	*F*-value/*t*-value	*p*-value
Sex				
Males	67 (51.1)	27.54 (8.19)	*t* = 2.75	0.007
Female	64 (48.9)	23.76 (7.65)		
Educational				
Primary school and below	17 (13)	19.26 (4.50)	*t* = 29.051	<0.001
Junior high	39 (29.8)	19.98 (4.41)		
High school or technical secondary school	37 (28.2)	30.06 (7.83)		
College, undergraduate and above education	38 (29)	30.24 (7.02)		
Types of insurance				
Private expense	43 (32.8)	26.01 (8.19)	*F* = 0.086	0.770
Medical insurance	88 (67.2)	25.56 (8.10)		
Family residence				
Rural district	59 (45)	19.62 (3.78)	*t* = −10.536	<0.001
Cities and towns	72 (55)	30.69 (7.26)		
Marital status				
Married	112 (85.5)	26.82 (7.92)	*t* = 4.072	<0.001
Single (including unmarried, divorced)	19 (14.5)	19.08 (5.67)		
Caregivers				
Spouse	100 (76.3)	25.20 (8.19)	*F* = −1.338	0.183
Children and others	31 (23.7)	27.45 (7.92)		
Fracture cause				
Direct violence	116 (88.5)	25.65 (8.19)	*F* = −0.173	0.863
Indirect violence and others	15 (11.5)	26.10 (7.92)		
Postoperative time (days)				
1–3	54 (41.2)	19.35 (4.41)	*t* = 74.879	<0.001
4–7	43 (32.8)	27.27 (7.38)		
Exceed 7	34 (26)	33.93 (4.32)		

### Correlation between personal mastery, self-efficacy and social support in patients after fracture surgery

As shown in Table 3, PMS scores were positively correlated with the GSES scores (*r* = 0.747, *p* < 0.01) and the PSSS (*r* = 0.299, *p* < 0.01).

**Table 3:** Correlation analysis of personal sense of control in patients after fracture surgery.

Variables	Personal mastery
Self-efficacy	0.747**
Social support	0.299**

*p < 0.05; **p < 0.01.

### Multiple linear regression analysis of personal mastery in patients after fracture surgery

Multiple linear regression analysis was performed with PMS scores of patients after fracture surgery as the dependent variable. Independent variables included statistically significant demographic characteristics (e.g., sex, education level, family residence, etc.) along with GSES and PSSS scores. The independent variables were assigned as follows, sex: male (X1 = 0, X2 = 0), female (X1 = 0, X2 = 1); standard of culture: primary school and below (X1 = 0, X2 = 0, X3 = 0), junior high school (X1 = 01, X2 = 1, X3 = 0), high school or technical secondary school (X1 = 1, X2 = 1, X3 = 0), college, bachelor’s degree or above (X1 = 0, X2 = 0, X3 = 1); family residence rural: (X1 = 0, X2 = 0), urban (X1 = 0, X2 = 1); marital status: married (X1 = 0, X2 = 0), single (including unmarried, divorced) (X1 = 0, X2 = 1); postoperative time (days): 1–3 days (X1 = 0, X2 = 0), 4–7 days (X1 = 1, X2 = 0), more than 7 days (X1 = 0, X2 = 1). The results of linear regression analysis showed that the patient’s place of residence, marital status, postoperative duration, and self-efficacy were important factors influencing the patient’s sense of personal mastery (Table 4) (*p* < 0.05). Specifically, married patients, families living in cities and towns, the longer the postoperative time, the higher the level of self-efficacy of fracture patients, the stronger the sense of personal mastery.

**Table 4:** Multiple linear regression analysis of personal mastery in patients after fracture surgery (*n* = 131).

Variables	*B*	SE	*β*	*t*-value	*p*-value	95% CI	
						Lower	Upper
Constant term	7.785	1.683		4.624	0.000	4.452	11.118
Sex (male as reference)							
Female	−1.027	0.593	−0.082	−1.733	0.086	−2.200	0.147
Standard of culture (primary school and below as a reference)							
Junior school	0.466	0.935	0.034	0.498	0.619	−1.386	2.318
High school or technical secondary school	1.679	1.100	0.120	1.525	0.130	−0.500	3.857
College, bachelor’s degree or above	0.815	1.141	0.059	0.714	0.476	−1.444	3.075
Family residence (rural as a reference)							
Cities and towns	2.610	0.781	0.206	3.341	0.001	1.063	4.157
Marital status (married as a reference)							
Single	−1.994	0.849	−0.112	−2.348	0.020	−3.675	−0.313
Postoperative time (1–3 days as a reference)							
4–7 days	2.858	0.804	0.213	3.556	0.001	1.267	4.450
More than 7 days	4.961	1.050	0.346	4.723	<0.001	2.881	7.040
Self-efficacy	0.376	0.063	0.400	5.988	<0.001	0.252	0.501
Social support	−0.016	0.031	−0.028	−0.522	0.603	−0077	0.045

*R*^2^ = 0.764; adjusted *R*^2^ = 0.75; *F* = 56.755; *p* < 0.001.

## Discussion

### The level of personal mastery of patients after fracture surgery needs to be improved

This study showed that the sense of personal mastery in patients after fracture surgery was 25.02 (6.3) points, representing a moderate level and slightly lower than that of patients with systemic lupus erythematosus (Drenkard et al., 2022). Among all items, “I can do almost anything I want to do” had the lowest score at 2.76 (1.11) points. This may be because patient’s physical strength remains relatively weak post-surgery. With the gradual wearing off anesthesia, limb pain gradually intensifies, causing patients to become nervous, fearful, and prone to psychological distress. Patients often fear that physical activity will exacerbate their pain, lowering patient expectations and reducing their autonomy and self-efficacy, which ultimately diminishes their sense of personal mastery (Bian et al., 2024). Medical staff should provide patients with professional treatment and rehabilitation guidance as early as possible to help them establish positive psychological coping styles. They can strengthen patients’ understanding of disease-related knowledge, alleviate fears in the early stages of illness, enhance their confidence, and improve their sense of personal mastery by organizing regular lectures and distributing disease-related.

### Patients living in cities and towns have a higher sense of personal mastery than those living in rural areas

This is corroborated by our research results, which show that the personal mastery scale scores of patients in urban areas were significantly higher than those of their rural counterparts (*p* < 0.05). We propose that this disparity can be largely attributed to the urban–rural gap in education levels, which directly impacts health literacy—the capacity to acquire and apply disease-related knowledge and skills (Jia et al., 2023). Specifically, higher educational attainment in urban areas equips patients with stronger abilities to access, understand, and utilize health information. This is supported by a recent cross-sectional study which identified lower education levels as a key factor explaining the rural–urban divide in health literacy (Deng et al., 2025). With enhanced health literacy, urban patients are better positioned to manage their daily health and respond effectively to emergencies. Crucially, the confidence gained from such effective management is directly linked to a greater sense of control over one’s life and health, a factor which has been shown to be strongly associated with improved patient outcomes (Heo et al., 2015). To bridge this gap, it is imperative for medical staff to tailor and strengthen health education for rural patients. Furthermore, primary healthcare institutions are vital in promoting disease prevention and management knowledge in rural communities, ensuring patients acquire the necessary skills and information to better manage their conditions.

### Married patients have a higher sense of personal mastery than single patients

Our results suggested that married patients had a higher sense of personal mastery than single patients (*p* < 0.05). This may be because spousal support provides positive emotional reinforcement, enhances self-worth, and reduces the perceived burden of illness. With emotional and practical support, married patients have a more positive attitude towards disease management and are more confident in managing their conditions, thus improving their quality of life and enhancing their sense of personal control (Bian et al., 2024). Accordingly, medical staff should pay greater attention to the needs of single patients, providing additional care, emotional support, and encouragement. By helping single patients adopt a positive outlook, medical staff can improve their confidence in managing their condition and enhance their sense of personal mastery postoperatively (Christie-Mizell et al., 2023).

### The longer the postoperative time of fracture patients, the stronger the sense of personal mastery

Our study showed that patients in the early postoperative period (1–3 days) had lower levels of personal mastery, likely due to severe pain. Conversely, patients in the later postoperative period (>7 days) exhibited higher levels of personal mastery. With an increase in postoperative time, the patient’s sense of personal mastery increased (*p* < 0.05). According to the analysis, patients could not adapt to their identity in the immediate postoperative period. Simultaneously, challenges such as local swelling of the affected limb, an unfamiliar environment, disease crisis, and other factors can aggravate the psychological pressure on these patients (Pavlović et al., 2024). They may develop fearful and skeptical attitudes towards the operation itself, as well as postoperative pain, infection, and recovery. These attitudes can lead to negative emotions, such as anxiety and depression, making it difficult for patients to adapt well to their illness and social life, ultimately affecting their quality of life (Song et al., 2020). Over time, however, patients can gather more information resources, enabling them to better understand their condition, adjust their attitudes towards the disease, adopt positive coping strategies, and build confidence in overcoming the disease. To support this process, medical staff should create a warm medical environment for patients and minimize sources of discomfort. They should help patients master pain management knowledge, such as teaching them how to use analgesic pumps and painkillers, and provide timely, accurate pain assessments in accordance with the three-step analgesia principle, in order to formulate a personalized analgesia plan for each patient. Additionally, it is crucial to monitor patients’ psychological well-being and implement intervention methods such as mindfulness exercise, decompression techniques, and relaxation training. These methods can help alleviate their fears, encourage patients to adopt positive coping styles to manage their condition, reduce their anxiety towards their illness, and improve their overall sense of personal mastery.

### Higher the level of self-efficacy in patients with fracture surgery, the stronger the sense of personal mastery

In this study, GSES scores for patients after fracture surgery averaged 24.6 (6.70), representing a moderate level. The results of multiple linear regression analysis showed that the level of self-efficacy could affect patients’ sense of personal mastery; the higher the level of self-efficacy, the higher the patient’s sense of personal mastery (*p* < 0.05). This is because such patients are likely to maintain a stable mood and a better psychological state, readily accept changes in their physical functions, and effectively regulate their negative emotions, as studies have shown (Jin et al., 2023). Higher general self-efficacy in postoperative patients can help form a positive coping style, help patients establish confidence, face the disease with a positive and optimistic attitude, actively cooperate with the medical staff for treatment, take the initiative to ask and start postoperative functional rehabilitation exercises, and actively seek the help of the nursing staff or family members in case of difficulties. Patients with high general self-efficacy (Wang et al., 2024) are more likely to choose positive coping measures and develop higher levels of personal mastery. Therefore, medical staff should actively improve patients’ self-efficacy, pay greater attention to patients’ daily behavioral habits after fracture surgery, supervise the implementation of health education, help and support patients to cultivate a positive lifestyle, promote the enhancement of general self-efficacy, and improve patients’ self-management of diseases, thus improving their level of personal mastery.

### Limitations

This study examined participants from a tertiary level-A hospital in Shanghai using a cross-sectional survey. The sample size was limited, representing only the results of a specific region at a particular time point. This study utilized a convenience sampling method at a single institution, which limits the external validity of our findings. Future multi-center studies employing random sampling and incorporating a large sample size are warranted to confirm and extend our results. Moreover, Previous research indicated that the sense of mastery serves as a mediating variable between social support and illness perception (Ding et al., 2023). That was, social support can influence patients’ illness perception by enhancing their sense of mastery, and improving this sense of mastery could help patients develop a more positive perception of their illness. The current study did not find a significant effect in this aspect, which might be attributed to the limited sample size and variations across different disease types. In addition, this study did not classify fracture types in detail, despite the variety of fractures. In the future, we can address limitations by increase the sample size categorizing fractures more precisely and conducting more specific, focused, and in-depth investigations to enhance the accuracy and applicability of findings.

## Conclusion

The results of this study identified that the personal mastery of patients after fracture surgery was at a medium level during hospitalization. Multiple linear regression analysis showed that family residence, marital status, postoperative time, and self-efficacy were significant influencing factors on personal mastery. Medical staff should dynamically pay attention to changes in patients’ personal sense of control after fracture surgery, formulate personalized nursing measures, implement strategies to enhance patients’ personal sense of control, accelerate the rehabilitation process, and improve patients’ quality of life.

## Data Availability

The raw data supporting the conclusions of this article will be made available by the authors, without undue reservation.
